# First report of *Cactodera milleri* Graney and Bird, 1990 from Colorado and Minnesota

**DOI:** 10.21307/jofnem-2021-017

**Published:** 2021-02-24

**Authors:** Andrea M. Skantar, Zafar A. Handoo, Mihail R. Kantor, Saad L. Hafez, Maria N. Hult, Kathryn Kromroy, Kimberly Sigurdson, Michelle Grabowski

**Affiliations:** 1 Mycology and Nematology Genetic Diversity and Biology Laboratory, USDA, ARS, Northeast Area, Beltsville, MD, 20705; 2 University of Idaho, Parma, ID, 83660; 3 Minnesota Department of Agriculture, Plant Protection Division, Saint Paul, MN, 55155

**Keywords:** Cyst nematode, *Quinoa*, Molecular markers, Hsp90, Ribosomal, Cytochrome oxidase I

## Abstract

In 2019, *Cactodera milleri* cysts were discovered from soil samples collected from a *Chenopodium quinoa* field, located in Mosca, Alamosa county, Colorado, USA. Approximately 200 lemon shaped cysts and several hundred juveniles were recovered from the affected quinoa plants. The same species was also identified from several counties in Minnesota from samples submitted over the years by the Minnesota Department of Agriculture as part of the Animal and Plant Health Inspection Service (APHIS) efforts to survey states for the presence of Pale Potato Cyst Nematode. The cysts and juveniles (J2) were recovered from soil samples through sieving and Baermann funnel extraction. The nematode species was identified by both morphological and molecular means as *Cactodera milleri* (Graney and Bird, 1990). To our knowledge this represents the first report of *Cactodera milleri* from Colorado and Minnesota.

The genus *Cactodera* (Krall and Krall, 1978) currently includes 16 species (Escobar-Avilia et al., 2020). *Cactodera milleri* (Graney and Bird, 1990) was originally described infecting the roots of common lambsquarter (*Chenopodium album*) in Michigan. Graney and Bird (1990) also performed a host range test which included 34 plant species (including weeds, agronomic crops, and cactus species). *Chenopodium quinoa* Willd. was among the species that were tested and identified as a host for *C. milleri* (Graney and Bird, 1990). This cyst nematode has a narrow distribution, being reported only in North America from Michigan (Graney and Bird, 1990), Wisconsin (Schroeder et al., 2008), and Indiana (Ferris et al., 1999). In November of 2019, lemon shaped cysts and juveniles were recovered from a soil sample collected from Alamosa county, Colorado, USA. The sample was sent to University of Idaho for identification (SH) who in turn forwarded the extracted cysts to the Mycology and Nematology Genetic Diversity and Biology Laboratory (MNGDBL) for identification purposes. In September of 2011, the Nematology Laboratory at Beltsville, MD analyzed and found *C. milleri* in a soil sample received from Mosca, Alamosa County, Colorado but at that time the presence of this nematode species in Colorado was not reported.

The Pale Potato Cyst Nematode National Survey program, which is an USDA program meant to keep under control the distribution of the Pale Cyst Nematode, is conducted nationwide by the States Department of Agriculture offices. The Minnesota Department of Agriculture is submitting regularly cysts samples to MNGDBL for identification purposes as part of the PCN program. Currently, *Cactodera milleri* is identified from seven different counties in Minnesota (Anoka, Wadena, Kittson, Todd, Clay, Polk, and Red Lake). In 2008, it was first identified in Wadena county and later from Kittson county, in 2011, 2015, and 2016.

## Materials and methods

### Various stages

Cysts, white females, second-stage juveniles (J2), and eggs were obtained from Colorado (CO) and Minnesota (MN). Juveniles for morphological observations were separated from soil by sieving and Baermann funnel extraction or were live recovered from cysts from fresh roots and kept in water in watch glasses. Juveniles were fixed in 3% formaldehyde and processed to glycerin by the formalin glycerin method (Golden, 1990; Hooper, 1970). Females and some cysts were typically removed from roots after fixation for 12 hr in 3% formaldehyde solution. Photomicrographs of cyst vulval cones, females, and J2 were made with an automatic 35-mm camera attached to a compound microscope having an interference contrast system. Photomicrographs of the specimens were made with a Nikon Eclipse Ni compound microscope using a Nikon DS-Ri2 camera. Measurements were made with an ocular micrometer on a Leica WILD MPS48 Leitz DMRB compound microscope. All measurements are in micrometers unless otherwise stated.

**Figure 1:**
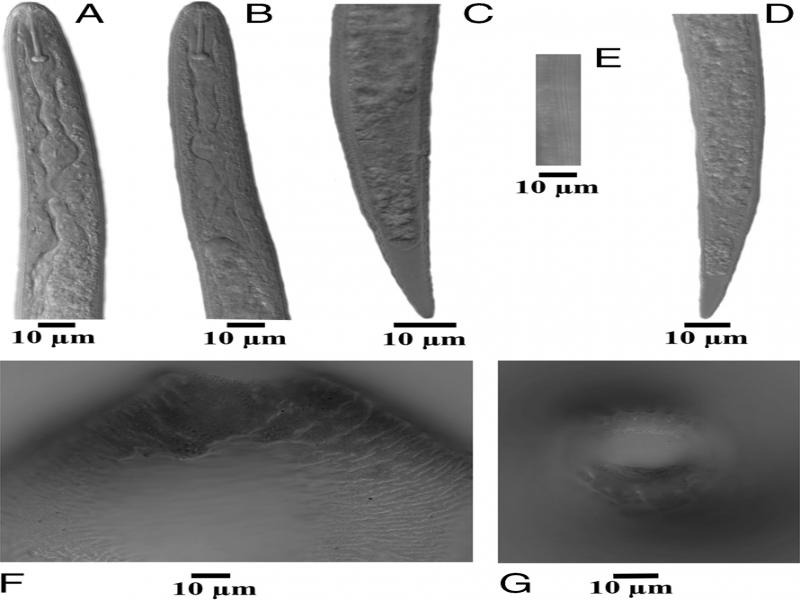
Photomicrographs of second-stage juveniles (J2) and cysts of *Cactodera milleri* from Mosca, Alamosa county, Colorado, USA. A, B: J2 anterior ends; C, D: J2 tails; E: J2 lateral field; F: Cyst posterior (ventral view); G: Cyst cone mount.

Living nematode juveniles (J2) recovered from the cysts were examined morphologically and molecularly for species identification. Observations of morphological characters critical for identification were: cyst shape, color and nature of fenestration, cyst wall pattern, J2 stylet length, shape of stylet knobs, and shape and length of tail and hyaline tail terminus (Fig. 1) thus indicated that the specimens were *Cactodera milleri*.

Representative J2 from two CO isolates and three MN isolates were used for molecular confirmation of the species, using two ribosomal genes (internal transcribed spacer, ITS 1 and 2 and large ribosomal subunit, 28S), one nuclear gene (partial heat shock protein 90, Hsp90), and one mitochondrial gene (partial cytochrome oxidase I, COI). Markers were amplified with the following primer sets: TW81 and AB28 for ITS rDNA; D2A and D3B for 28S rDNA; U288 and L1110 for Hsp90; and primers JB3 and JB4.5 for COI (Table 1). DNA extraction, amplification, purification of PCR products, cloning, and sequencing were performed as described in the studies of Skantar et al. (2012) and Subbotin et al. (2017). DNA sequencing was conducted by University of Maryland Center for Biosystems Research and Genewiz, Inc. ITS rDNA and Hsp90 sequences were obtained from cloned amplicons; 28S and COI were sequenced directly from PCR products.

**Table 1.** Primers used for molecular analysis of *Cactodera *spp.

Primer	Sequence (5′→3′)	Marker	References
D2A	ACAAGTACCGTGAGGGAAAGTTG	28S rDNA	De Ley et al. (2005)
D3B	TCGGAAGGAACCAGCTACTA		
TW81	GTTTCCGTAGGTGAACCTGC	ITS rDNA	Joyce et al. (1994)
AB28	ATATGCTTAAGTTCAGCGGGT		
JB3	TTTTTTGGGCATCCTGAGGTTTAT	COI mtDNA	Bowles et al. (1992)
JB4.5	TAAAGAAAGAACATAATGAAAATG		
U288	GAYACVGGVATYGGNATGACYAA	Hsp90	Skantar and Carta (2004)
L1110	TCRCARTTVTCCATGATRAAVAC		

New sequences were submitted to GenBank under the following accession numbers: ITS from CO (MT327799, MT327800) and MN (MK619682-MK619693); Hsp90 from MN (MK105547-MK105550) and CO (MT362485-MT362487, MN182650-MN182651); COI from CO (MT328830-MT328835); and 28S from MN (MK619472, MK619473), and CO (MT328175, MT328180-MT382182).

**Figure 2:**
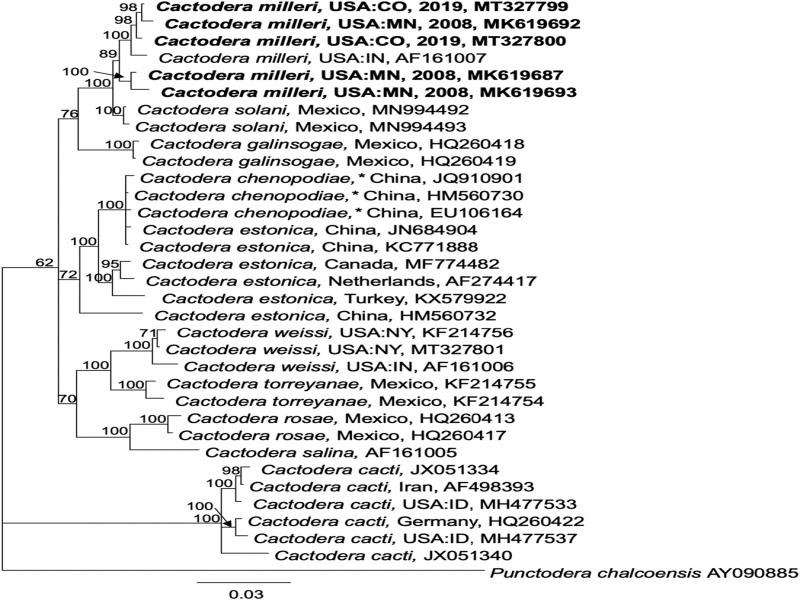
Phylogenetic relationships of *Cactodera milleri* from CO and MN with other select cyst nematodes, as inferred from a 995 bp alignment of ITS 1 and 2 rDNA, according to the GTR + I + G model of nucleotide substitution and incorporated into MB as described. A 50% majority rule consensus tree was generated with posterior probabilities (PP) shown on appropriate branches (expressed as percent), with *Punctodera chalcoensis* as the outgroup. New sequences are indicated in bold. *Cactodera chenipodiae* marked with an asterisk (*) reflect corrected identifications as described in Escobar-Avilia et al. (2020).

**Figure 3:**
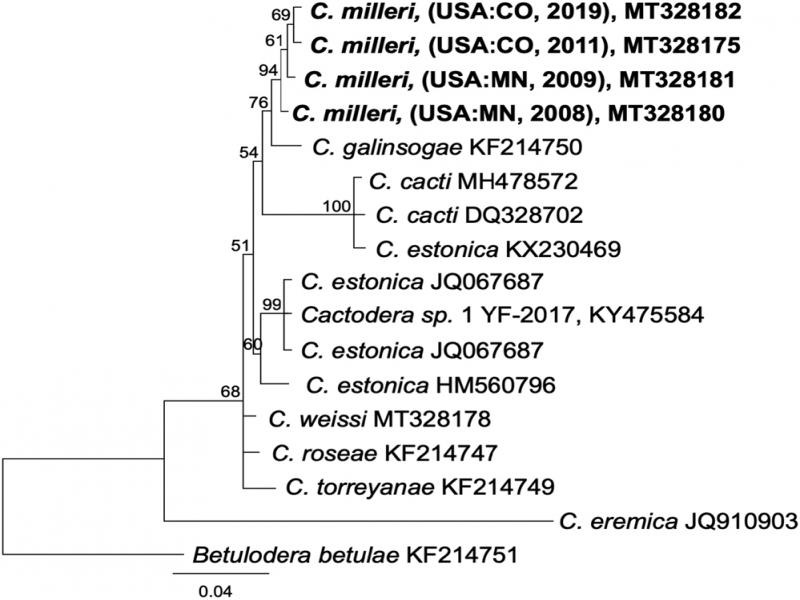
Phylogenetic relationships of *Cactodera milleri* from CO and MN with other select cyst nematodes, as inferred from a 659 bp alignment of 28S rDNA, according to the GTR + I + G model of nucleotide substitution and incorporated into MB as described. A 50% majority rule consensus tree was generated with posterior probabilities (PP) shown on appropriate branches (expressed as percent), with *Betulodera betulae* as the outgroup. New sequences are indicated in bold.

**Figure 4:**
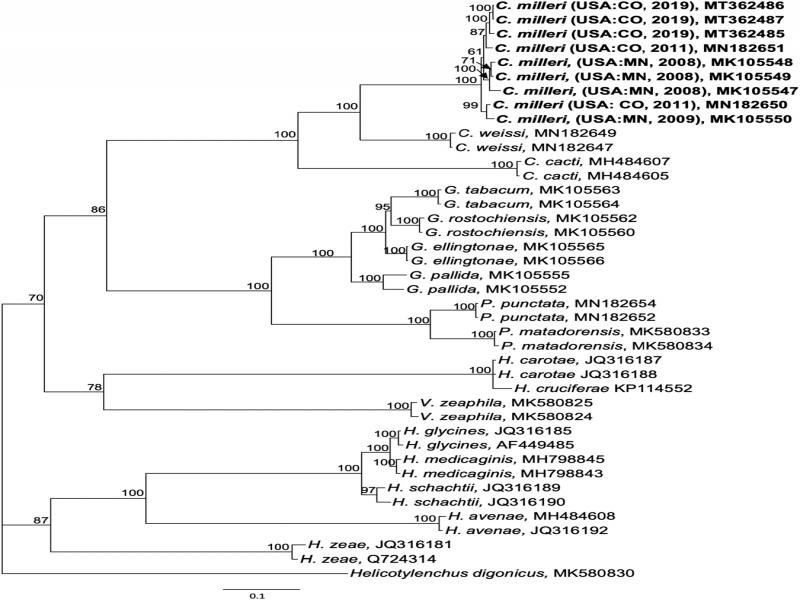
Phylogenetic relationships of *Cactodera milleri* from CO and MN with other Heteroderidae, as inferred from a 2340 bp alignment of Hsp90 genomic DNA, according to the GTR + I + G model of nucleotide substitution and incorporated into MB as described. A 50% majority rule consensus tree was generated with posterior probabilities (PP) shown on appropriate branches (expressed as percent), with *Helicotylenchus digonicus* as the outgroup. New sequences are indicated in bold.

Separate alignments of ITS rDNA, Hsp90, and COI sequences were constructed using the MAFFT algorithm within Geneious v. 10.2.6 (Biomatters, Ltd., San Diego, CA). For ITS, the best-fitting model of nucleotide substitution, General Time Reversible with Gamma distributed rates with Invariant sites (GTR + I + G), was estimated using jModelTest based on the Akaike information criterion. Phylogenetic relationships were estimated by Bayesian inference (BI) on the CIPRES Science Gateway (http://www.phylo.org; Miller et al., 2010) plug-in within Geneious, with *Punctodera chalcoensis* (AY090885) set as the outgroup. Markov chains were sampled at intervals of 500 generations and burn-in of 5,000. A 50% majority rule consensus tree was generated with posterior probabilities (PP), expressed as a percent, calculated for each clade (Figs. 2-4). 28S rDNA alignments were analyzed in the CIPRES MrBayes under the model GTR + I + G, with *Betulodera betulae* (KF214751) as outgroup. Hsp90 genomic alignments were analyzed under the model GTR + I + G implemented in MrBayes as described above, with *Helicotylenchus digonicus* (MK580830) set as the outgroup. COI mtDNA DNA alignments were analyzed in CIPRES under the model GTR + I + G implemented in MrBayes as described above, with *Rhizonemella* sp. (MF425746) as the outgroup. Alternative trees for each marker were made using the maximum likelihood tree-building algorithm IQ-tree (Nguyen et al., 2015) or the RaXML CIPRES module within Geneious (not shown).

**Figure 5:**
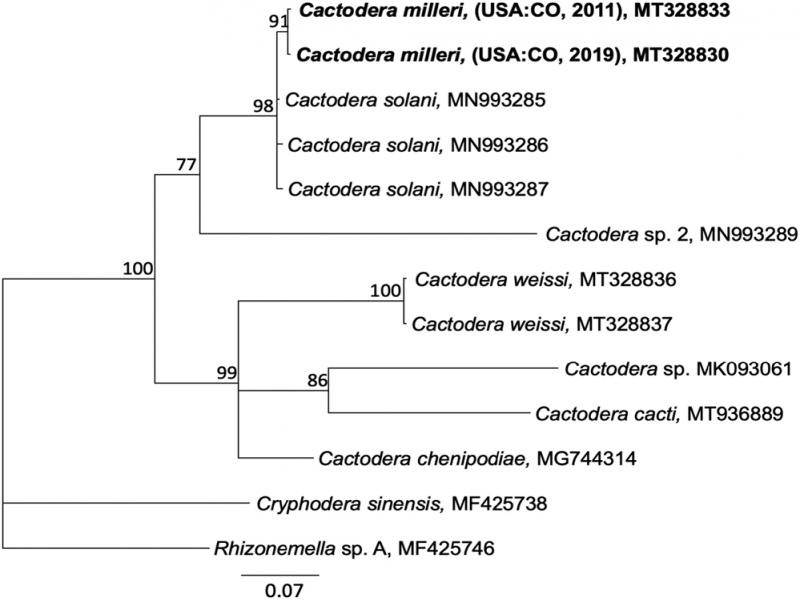
Phylogenetic relationships of *Cactodera milleri* from CO with other select *Cactodera* species nematodes, as inferred from a 358 bp alignment of mitochondrial COI, according to the GTR + I + G model of nucleotide substitution and incorporated into MB as described. A 50% majority rule consensus tree was generated with posterior probabilities (PP) shown on appropriate branches (expressed as percent), with *Rhizonemella* sp. and *Cryphodera sinensis* as the outgroups. New sequences are indicated in bold.

## Description

### Measurements

Measurements of second-stage juveniles from Colorado (*n* = 15) included length of body (range = 427-490 μm, mean = 459.6 μm), stylet well developed (21.0-23.5 μm, 22.4 μm) with concave basal knobs (slight to deeply concave), tail (38.0-45.0 μm, 40.73 μm), and hyaline tail terminus (14.0-20.0 μm, 16.76 μm). The lateral field had four distinct lines. Shapes of the tail, tail terminus, and stylet knobs were also consistent with *C. milleri.* The cysts (*n* = 10) were lemon shaped, abullate, circumfenestrate, dark brown in color and had a straight to wavy line type of cyst wall cuticular pattern in the middle (Fig. 1F, G). The fenestra diameter ranged between 25 and 35 μm, with a mean 30.02 μm. Morphometrics of cysts were also consistent with *C. milleri*. Morphometrically, it is also close to *Cactodera solani* (Escobar-Avilia et al., 2020) in having small rounded to lemon shaped cysts with a circumfenestrate vulval cone and in the J2 shape and length of tail and tail terminus. However, it differs from *C. solani* by having longer cysts 632 μm (515-730 μm) vs 417 μm (291-581 μm), cysts light to dark brown vs light brown to almost black in color, and J2 stylet length being shorter 22 μm (21-23 μm) vs longer stylet length 25 μm (24-27 μm). In addition, *C. solani* is the only species in the genus known to parasitize tomato.

### Molecular analysis

The ITS rDNA clone sequences from the CO population varied 2 bp from each other, while differences among MN population clones ranged from 4 to 14 bp. The exceptions were one MN clone (MK619692) and the *C. milleri* sequence from Indiana (AF161007) which contained 8 bp and 7 bp deletions (at different locations) in addition to single base polymorphisms throughout the sequence. Intraspecific variation among all available ITS sequences from *C. milleri* ranged from 0.2 to 3.5%. The next closest species was *C. solani*, which had lower intraspecies variation of 0 to 2 bp (0-0.1%). *Cactodera milleri* differed from *C. solani* at 7 to 24 bp. In phylogenetic trees, *C. milleri* formed a strongly supported clade distinct from *C. solani* from Mexico (Fig. 2), consistent with data shown previously in the *C. solani* description (Escobar-Avilia et al., 2020).

28S rDNA sequences from *C. milleri* (CO, 2019) were identical to an isolate obtained in 2011 and 2 bp different from MN isolates. In the MB tree (Fig. 3), *C. milleri* formed a clade separate from the next closest available species, *C. galinsogae* (KF214147), differing at 8 to 10 bp (Fig. 2). No *C. solani* sequences were available for direct comparison.

Partial Hsp90 sequences were aligned with selected sequences from other *Cactodera* spp. and other cyst nematodes as available. Hsp90 sequences from the CO and MN populations varied at 4 to 22 bp (0.3-1.7%), primarily in introns, except for one clone from MN (MK105547) that contained a 56 bp shorter intron than the other sequences. Based upon Hsp90 sequence alignment, *C. milleri* formed a strongly supported clade that was distinct from *C. weissi*, *C. cacti*, and other cyst nematodes (Fig. 4).

COI sequences from 2011 and 2019 CO isolates were identical to each other and varied from *C. solani* at 3 to 4 bp (0.9-1.2%). In the tree based upon COI alignments, these two species formed separate clades with moderate support. Relatively few COI sequences are available in GenBank, so contribution of additional sequences representing *Cactodera* species will undoubtedly contribute to an improved understanding of the genus (Fig. 5).

Based upon this collective morphological and molecular data, we identify this isolate as *Cactodera milleri.* The closest species morphologically and molecularly is *C. solani*, but was distinguishable based upon direct comparison of ITS rDNA and COI. To our knowledge this is the first report of the *Cactodera milleri* in Colorado as well as in Minnesota. Together, these data contribute to a growing picture of the phylogenetic relationships within the Heteroderidae, with Hsp90 and COI complementing widely used ribosomal markers for identification of species within the genus *Cactodera*. The amplified region of Hsp90 contains a relatively high degree of polymorphism, particularly within the introns, providing a rich source of intraspecific variation upon which to separate species. Coding regions or translated protein sequences also contain information that may prove beneficial for phylogenetic analysis of circumfenestrate nematodes (Skantar et al., 2019, 2020).
